# Targeting the Tumor Microenvironment in Neuroblastoma: Recent Advances and Future Directions

**DOI:** 10.3390/cancers12082057

**Published:** 2020-07-25

**Authors:** Shweta Joshi

**Affiliations:** Department of Pediatrics, Division of Pediatric Hematology-Oncology, Moores Cancer Center, University of California San Diego, La Jolla, CA 92093-0815, USA; shjoshi@health.ucsd.edu; Tel.: +1-858-822-7580

**Keywords:** tumor microenvironment, neuroblastoma, immunosuppression, hypoxia, extracellular matrix, metastasis

## Abstract

Neuroblastoma (NB) is the most common pediatric tumor malignancy that originates from the neural crest and accounts for more than 15% of all the childhood deaths from cancer. The neuroblastoma cancer research has long been focused on the role of MYCN oncogene amplification and the contribution of other genetic alterations in the progression of this malignancy. However, it is now widely accepted that, not only tumor cells, but the components of tumor microenvironment (TME), including extracellular matrix, stromal cells and immune cells, also contribute to tumor progression in neuroblastoma. The complexity of different components of tumor stroma and their resemblance with surrounding normal tissues pose huge challenges for therapies targeting tumor microenvironment in NB. Hence, the detailed understanding of the composition of the TME of NB is crucial to improve existing and future potential immunotherapeutic approaches against this childhood cancer. In this review article, I will discuss different components of the TME of NB and the recent advances in the strategies, which are used to target the tumor microenvironment in neuroblastoma.

## 1. Introduction

Neuroblastoma is an extremely heterogeneous pediatric tumor that arises from neural crest and patients with this malignancy account for more than 15% of all childhood cancer deaths [1,2]. The overall incidence of neuroblastoma is 1 patient per 100,000 children and every year 600–700 new cases are diagnosed in the United States of America [3]. NB patients are stratified into low-, intermediate- and high-risk groups, based on different parameters, including tumor histology, clinical stage, tumor cell ploidy, and MYCN oncogene amplification [4]. In approximately, 50% of children with this disease, tumors lack amplification of MYCN oncogene and these patients show overall survival more than 90%. However, the other half of the patients constitutes a high-risk group and these patients either harbor tumors with MYCN amplification, or are older than 18 months and display metastatic disease regardless of MYCN amplification. Despite the most advanced and intensive therapeutic approaches that combine surgery, myeloablative chemotherapy, radiation therapy, and anti-disialoganglioside (GD2) mAb ch14.18 based immunotherapy, the chances of long term survival in the high-risk group is even less than 40% [5,6]. Hence new and effective therapeutics are required to treat patients with high-risk NB.

The development of effective anti-cancer therapies is highly challenging due to the complex nature of tumor microenvironment [7,8]. Tumor microenvironment (TME) is a highly specialized niche that develops during tumor progression and is comprised of not only tumor cells, but vascular endothelial cells, cancer associated fibroblasts (CAFs), mesenchymal stromal cells (MSCs), Schwann cells and infiltrating immune cells (T cells, B cells, tumor associated macrophages, myeloid derived suppressor cells, natural killer cells) are also major components of TME. The initiation and progression of tumor growth rely on complex series of biological events in a normal cell that leads to uncontrolled cell proliferation, resistance to apoptosis, alteration of tumor cell metabolism, and remodeling of extracellular matrix [9,10,11]. These events are orchestrated by communication between stromal and immune cells, which further promote inflammation, stiffness of extracellular matrix (ECM), maturation of TME, vascular remodeling and metastasis [12]. TME plays a crucial role in tumor progression, metastasis, immunosuppression and resistance of tumor cells to chemotherapy and checkpoint inhibitor therapy. Hence, the remodeling of TME has recently emerged as a novel strategy in treating solid cancers, including NB [7,13,14].

In this review article, I will discuss different components of TME of neuroblastoma, with a special focus on targeting extracellular matrix, hypoxia, tumor vasculature, stromal cells and infiltrating immune cells. I will also discuss the recent ongoing clinical trials in neuroblastoma patients that target TME and will review future directions for targeting TME in NB.

## 2. Genetic Alterations and Tumor Microenvironment of Neuroblastoma

Studies undertaken to evaluate and understand the TME of NB is highly challenging due to the limited availability of primary tumors with viable cell populations. Patients with high-risk disease are treated with various cycles of chemotherapy followed by surgical removal and at this time most of the tumors get necrotic and calcified, and these tumors cannot be used for studies requiring viable cells. However, recently, molecular techniques have allowed the gene expression profiling of NB tumors in depth and these studies have provided information on the improved treatment stratification of NB and TME of neuroblastoma [15,16,17]. These studies have shown that nearly 20% of NB patients show amplification of MYCN oncogene while non-MYCN amplified NB cases display other chromosomal rearrangements such as 3p, 4p, 11q loss or 1q, 2p, and 17q gains. Pugh et al., has studied the spectrum of somatic mutations in high risk neuroblastoma and reported low exonic mutation frequency and very few recurrently mutated genes in these tumors [18]. Genes with significant somatic mutation frequencies reported according to this study included ALK, PTPN11, ATRX, MYCN, and NRAS.

Various signaling pathways, including PTEN/PI3K/AKT and RAF/MEK/ERK control stabilization of MYCN and are major mediators of uncontrolled tumor growth, angiogenesis, invasion, apoptosis and cellular metabolism in neuroblastoma [19,20,21]. PI3K/AKT signaling axis controls GSK3β dependent regulation of MYCN and stabilization of HIF1α and hence the efficacy of inhibitors targeting these signaling axes has been tested in NB models [22,23,24]. Seeger and his group have extensively studied non-MYCN amplified tumors and have provided valuable information on the TME of these tumors [25,26,27]. A recent study by this group revealed that metastatic tumors had higher infiltration of tumor associated macrophages (TAMs), as compared to loco-regional tumors in non-MYCN amplified NB tumors [28]. This study has also identified that neuroblastoma patients with an age of ≥18 months had higher expression of inflammation-related genes (IL10, IL6R, CD16, CD33, and FCGR3), as compared to patients diagnosed at age ≤ 18 months. TAMs also contribute to the stimulation of hypoxic microenvironment in NB by inducing transcription of hypoxia inducible factor (HIF 2α) [29]. Besides macrophages, the presence of cancer associated fibroblasts (CAFs), mesenchymal stromal cells (MSCs), endothelial cells and inflammatory immune cells including regulatory T cells (T_reg_), myeloid derived suppressor cells (MDSCs); also contributes to highly vascular, angiogenic, hypoxic and immunosuppressive microenvironment of NB [7,30,31,32]. Immunosuppressive microenvironment in NB is generated due to various reasons, which include (1) infiltrating immunosuppressive immune cells including macrophages, regulatory T cells and myeloid derived suppressor cells, (2) soluble factors secreted in neuroblastoma microenvironment which mediates immunosuppression like TGF beta, IL10, and galectin-1, (3) defects in antigen-presenting machinery (APM) and low levels of MHC class I molecule displayed by NB cells. The details on immunosuppressive microenvironment have been reviewed in detail before [30,31].

## 3. Targeting the Tumor Microenvironment of NB

**Figure 1:**
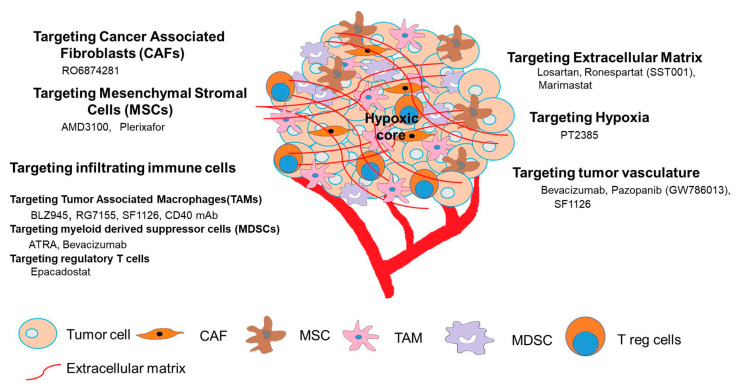
Strategies used to target tumor microenvironment in neuroblastoma.

Cancer research has long been focused on targeting the tumor cells only and monoclonal antibodies targeted against GD2, a ganglioside present selectively in human NB tumor cells has shown great promise in NB patients, especially in the setting of minimal residual disease [33,34,35]. Several clinical trials are ongoing with murine or chimeric anti-GD2 monoclonal antibodies either alone or in combination with IL2, GMCSF and retinoic acid [5,36,37]. Clinical trial with ex-vivo expanded and activated donor natural killer cells with Hu14.18-IL2 is also ongoing for patients with relapsed or refractory neuroblastoma (NCT03209869). Recently genetic engineering of T lymphocytes to express anti-GD2 chimeric antigen receptor (CAR) has also been developed and tested in clinical trials [38,39,40]. However, despite the clinical success of CAR T cells in hematological malignancies, the efficacy of this therapy has not shown any significant benefit in solid tumors including NB due to immunosuppressive TME in neuroblastoma [30,41]. Hence, understanding the TME of NB is crucial for the generation of efficient therapeutic strategies to treat this childhood cancer. Here, we describe the current strategies used to target the TME of NB, as shown in Figure 1.

### 3.1. Targeting the Extracellular Matrix

The extracellular matrix is a three-dimensional network of extracellular molecules, composed of collagen, elastin, fibronectin, reticulin fibers, hyaluronic acid, proteoglycans, and glycoproteins that provides both structural and biochemical support to surrounding cells, and is responsible for transmitting extracellular signals to cells [42]. The composition of ECM plays an important role in the progression of the tumor by promoting angiogenesis, invasion, and metastasis [42]. The heterogeneity of tumor cells, hypoxia and augmented inflammation in the TME promote alterations in the ECM components which lead to increased collagen deposition and increased ECM density and stiffness [43]. In NB, the collagen fiber matrix augments the differentiation of human neural crest stem cells towards Schwann cell lineage and increased collagen cross-linking contributes to tumor progression [44,45]. In addition to this, reticulin fibers, which are composed of collagen III fibers influence morphological changes of the cell as well as affect biological functions in NB [46]. The rigidity or stiffness of ECM also affects the cellular behavior of NB cells including stem cell differentiation, neurite extension, proliferation, and malignant potential of these cells [47,48,49]. Several studies have shown that alterations in the biochemical composition of ECM promote morphological differentiation of NB cells [50,51,52]. Lam et al. have shown that rigidity of ECM increases neuroblastoma cell differentiation and N-Myc expression [53]. In addition, various authors have shown that increasing stiffness of ECM can augment neuritogenesis, inhibit proliferation of neuroblastoma cells, reduce the expression of N-Myc and addition of retinoic acid can enhance these effects. The role of glycosaminoglycans in cell anchoring and scaffolding properties has been studied in some malignancies including NB and they are recently considered as novel therapeutic targets in these malignancies [54,55,56]. In a study by Irene Tadeo, the authors used advanced morphometric approaches to evaluate blood vessels, elastic fibers, reticulin fiber networks, collagen type I bundles and glycosaminoglycan in 102 high-risk NB samples [57]. Based on the organization of reticulin fibers and blood vessels, authors have found that 30 out of 102 patients belongs to ultra-high risk group within this high-risk group and these patients have a 5-year survival rate of <15%. This ultra-high risk group is classified on the basis of death of patients from this disease within 18 months after diagnosis. Neuroblastoma tumor cells also express several integrins to communicate with the ECM [58]. Integrin α_4_β_1_ is reported to enhance metastasis and is associated with poor prognosis in non MYCN amplified tumors [59]. Some studies have shown that integrin α_v_β_3_ is expressed on endothelium was increased in high metastatic, stage 4 NB as compared with localized NB [60]. In another report, α_v_β_3_ integrin is reported to be expressed on 68% of microvessels in MYCN amplified stage 3 neuroblastoma and 34% in MYCN-non amplified tumors [22].

To target ECM, angiotensin receptor antagonist, Losartan, has shown great effects in reducing secretion of collagen I and in improving the delivery of chemotherapeutic drugs [61]. Ronespartat (SST001), an heparanase inhibitor has shown great efficacy in inhibiting tumor growth of human pediatric sarcoma models either alone and in combination with anti-angiogenic agents [62]. The role of matrix metalloproteinase (MMPs) in the modulation of ECM in TME and its association with poor prognosis in NB is also well-documented [63,64,65]. MMPs promote the degradation of ECM barriers and release active growth factors and promote cancer cell invasion and tumor angiogenesis [66]. Several MMP inhibitors, such as Marimastat, Incyclinide (CMT3, COL 3), and Minocycline, are developed that went into clinical trials for advanced carcinomas [67,68]. One pre-clinical study has shown that Marimastat reduced the in vitro invasion of neuroblastoma cells [69]. Other MMP targeting strategy includes highly selective MMP9 inhibitor JNJ0966 that allosterically inhibit zymogen activation [68]. In another report, MMP14 targeting antibody Fab 3369 blocks immunosuppression and metastasis in triple-negative cancer [70]. However, most MMP targeting inhibitors did not show promising results in clinical trials with respect to other cancers [71], and hence their application in early cancer stages can provide some benefit in clinical trials. These inhibitors have rarely been tested for targeting MMP in pediatric neuroblastoma and hence should be explored in the future.

### 3.2. Targeting Hypoxia

The rapid proliferation of tumor cells leads to an increase in the requirement of oxygen supply which cannot be fulfilled by surrounding blood vessels, resulting in limited oxygen supply to the cells and hypoxia [72]. Hypoxia initiates a series of cellular responses in reaction to a low oxygen supply, mainly coordinated by transcription factors, hypoxia inducible factor 1 and 2 [73]. These transcription factors are known to regulate genes involved in glucose metabolism, cell proliferation, angiogenesis and polarization of tumor associated macrophages [10,73]. The expression level of both HIF 1 and 2 are known to correlate with patient outcome in various solid cancers, including neuroblastoma [29,74,75]. In NB, the expression of HIF 1 and HIF 2 α has revealed interesting differences with disease stage and clinical outcome [76]. A study by Pietras et al. has shown that fast growing tumors show high immunostaining of HIF factors in high-risk neuroblastoma. Studies by Jogi et al. and Fredlund et al. have shown that hypoxia promotes aggressive neuroblastoma features [77,78]. These studies have shown positive correlation between hypoxia and immature neuroblastoma phenotype [77] and its association with poor clinical outcome [78]. HIF1α expression is associated with the low tumor grade and favorable prognosis while expression of HIF2α correlated with high tumor grade and unfavorable prognosis [76,79,80]. These studies illustrate that tumor cells that stained positive for HIF2α are more aggressive and are linked to high-risk disease. In another study by Dungwa et al., the authors have reported that HIF1α levels correlate positively with MYCN amplification and various other adverse prognostic factors like 1p deletion and 17q gain [81]. In this study, authors have found significant decrease in event free survival and overall survival when immunoexpression of HIF-1α is considered positive for ≥ 10% of tumour cells. The main finding of this study is that expression of HIF-1α is high in aggressively growing NB tumors. In another study by Applebaum et al., authors have found a set of genes regulated by hypoxia and the expression of these genes correlate well with adverse outcome in neuroblastoma patients [82]. Pietras et al. have shown that HIF 2α maintains human NB cells in an undifferentiated state and hence targeting HIF2α is an effective strategy to treat NB [83]. VEGF is a downstream target of both HIF1α and HIF2α and the expression level of both isoforms correlate positively with VEGF expression in NB [76]. Various reports have demonstrated HIF2α is an attractive therapeutic target in neuroblastoma [79,83]. PT2385 a novel inhibitor of HIF2α transcriptional activity has been tested in a preclinical model of clear cell renal cell carcinoma [84,85]. PT2385 and its analog PT2399 have shown great efficacy in preclinical models of clear cell renal cell carcinoma (ccRCC) [84,86], which leads to phase 1 clinical trial of this molecule in patients of advanced ccRCC (NCT02293980). The effect of PT2385 has been evaluated in only one study in neuroblastoma and the study has shown that PT2385 did not affect the cellular response to chemotherapy in neuroblastoma PDX model [87]. However, recently no clinical trial of this drug is in place for neuroblastoma patients, but this drug can be evaluated in NB clinical trials, based on the efficacy of this drug in ccRCC and recurrent glioblastoma.

### 3.3. Targeting Tumor Vasculature

The work from various labs indicate that vascularization of the tumor is highly coordinated and regulated by a physiological response to hypoxia and inflammation [88,89]. The initiation of angiogenesis also known as “angiogenesis switch” is induced by the release of pro-angiogenic factors [90]. VEGF/VEGFA is the most common pro-angiogenic factor found in neuroblastoma tumors, but many other factors like fibroblast growth factor (FGF), platelet-derived growth factor (PDGF) are reported in NB [91,92,93]. In neuroblastoma, tumor vasculature is associated with an aggressive phenotype [94,95,96]. The over-expression of VEGF has been demonstrated in neuroblastoma and preclinical studies have shown that expression of VEGF correlates with the high-risk disease in NB [97,98]. In a recent study, Jakovljevic et al. have determined VEGF expression in paraffin- embedded primary tumor tissue from 56 neuroblastoma patients and reported that VEGF expression correlated with disease stage in NB patients [99]. There is also evidence that PI3K up-regulates the expression of VEGF via MYCN dependent mechanisms in NB and the use of PI3K/mTOR inhibitors suppresses NB tumor progression by regulating MYCN degradation, and through paracrine blockade of angiogenesis [20,100]. Various pre-clinical studies have shown that anti-angiogenic strategies might be effective in NB [101,102,103]. Bevacizumab is an anti-human VEGF antibody and is used to treat various cancers, including neuroblastoma [104,105]. Several clinical trials of bevacizumab both alone or in combination with various other agents are completed or ongoing in neuroblastoma and are shown in Table 1. In 2008, a phase I clinical trial was opened to explore the maximum tolerated dose of bevacizumab in pediatric patients with refractory relapsed tumors [106]. This study has shown that bevacizumab is well-tolerated in children, which leads to the opening of phase 2 clinical trials of bevacizumab in combination with other chemotherapeutic drugs. In a phase II study bevacizumab, is paired with irinotecan, and temozolomide for refractory and relapsed NB patients (NCT01114555) [107]. The combination of drugs was well tolerated but the addition of bevacizumab did not improve response rates over a combination of irinotecan and temozolomide [107]. Bevacizumab has shown great results in the preclinical neuroblastoma model in combination with cyclophosphamide [108]. Based on these pre-clinical studies, a phase II clinical trial of Bevacizumab with cyclophosphamide and topotecan is completed in patients with relapsed/refractory Ewing sarcoma and neuroblastoma (NCT01492673). In a separate Phase 1 clinical trial, cyclophosphamide and Bevacizumab are combined with zoledronic acid for treatment of patients with recurrent or refractory high-risk neuroblastoma patients (NCT00885326). Bevacizumab has also been combined with radioimmunoconjugate consisting of 3F8, a murine anti-GD2 antibody labeled with iodine 131 (I-131) in phase I clinical trial for treating patients with relapsed or refractory neuroblastoma (NCT00450827) [109]. In another ongoing Phase 2 neuroblastoma randomized clinical trial (NCT02308527) also known as BEACON neuroblastoma trial, bevacizumab, irinotecan, and temozolomide has been combined with topotecan for treatment of patients with relapsed or refractory neuroblastoma [110]. A recent study has shown that bevacizumab treatment can improve the anti-tumor efficacy of GD2-CAR cells in the human neuroblastoma preclinical model [111]. Pazopanib (Votrient) is a multi-kinase inhibitor and has shown anti-angiogenic activity in combination with topotecan in pediatric solid tumors [112,113]. A phase I study of Pazopanib was initiated to study the efficacy of this agent in refractory pediatric neuroblastoma tumors (NCT01130623).

The pre-clinical studies carried out in our lab have shown that dual PI3K/BRD4 inhibitors SF1126 and SF2523 suppress neuroblastoma tumor growth, angiogenesis and metastasis [22,114]. Both SF1126 and SF2523, orthogonally hit PI3K and BRD4 signaling, which blocks MYCN expression, activation and promotes MYCN degradation, ultimately leading to reduced tumor growth, angiogenesis and tumor metastasis [22,114,115]. BRD4 inhibitors are known to inhibit transcription of MYCN, induce apoptosis and impair tumor growth of neuroblastoma [116]. PI3K inhibitors are also reported to kill neuroblastoma cells by inducing degradation of MYCN [117]. Hence the effect of SF2523 on tumor angiogenesis and metastasis are secondary effect of this drug on TME. SF1126 also blocked tumor angiogenesis, metastasis, and increased the M1 to M2 ratio in various preclinical mouse models, including neuroblastoma [118,119,120,121], which lead to phase 1 clinical trial of this drug in various solid tumors (NCT00907205) and pediatric neuroblastoma malignancies (NCT02337309). Some studies have shown that fibronectin isoform, B-FN is a marker of angiogenesis [122,123] and targeting tumor vasculature using L19 (scFv), a human recombinant specific antibody specific for B-FN, has provided benefit in cancer patients and experimental mouse models [124,125]. In another study, Balza et al., has shown that targeting TNF α and IL2 to NB cells by L19 (scFv), can cure and vaccinate animals and is strongly associated with the generation of adaptive immunity involving CD4+ and CD8+ T cells in neuroblastoma model [126]. L19-TNFα and L19-IL2 have been used in preclinical models of cancer and are presently undergoing testing in phase I/II clinical trials for cancer treatment [127].

### 3.4. Targeting Stromal Cells

Solid tumors contain malignant cells, as well as different kinds of stromal cells, which includes immune cells, endothelial cells, cancer associated fibroblasts (CAFs) and mesenchymal stromal cells (MSCs). The interplay between tumor cells and stromal cells contributes to tumor progression and metastasis. In this section, I will discuss about CAFs and MSCs and immune cells will be discussed in next section.

#### 3.4.1. Targeting Cancer Associated Fibroblasts

As cancer progresses, fibroblasts get converted to CAFs and these activated fibroblasts share similarities with fibroblasts activated during wound healing [128]. CAFs acquire features of myofibroblasts, including the increased production of α-smooth muscle actin (α SMA) and promote tumor growth and progression. CAF infiltration has been associated with poor clinical outcomes in various cancers including neuroblastoma [129,130,131]. CAFs secrete TGFβ and SDF-1/CXCL12 that regulate EMT transition and recruit endothelial progenitor cells to the tumor site to facilitate angiogenesis, and tumor growth, respectively [132,133]. TGFβ is a cytokine which also mediates immunosuppression in the TME [134]. In addition, CAFs secrete cytokines such as CCL2 and SDF to recruit TAMs in the TME [133]. The role of CAFs in the progression of neuroblastoma has been studied in 60 NB tumors and this study has found a high number of CAFs correlate with microvessel density [131]. This study has also shown that CAF inversely correlates with Schwannian stroma in neuroblastoma tumors and authors have suggested that Schwann cells might prevent the activation of CAFs. A recent study by Hashimoto et al. has shown that TAMs and CAFs closely interact in the TME and this interaction provides a favorable environment for neuroblastoma progression [135]. This study has also shown that both, the number of TAMs and the area of CAFs were significantly correlated with clinical stage and MYCN amplification. Recent studies have shown that CAFs also contribute to immunosuppressive TME by secreting TGFβ, and hence, CAFs are excellent therapeutic targets for immunotherapy approaches for cancer [136]. In pre-clinical studies, the TGFβ2 antisense modified allogeneic tumor cell vaccine showed increased efficacy in an intracranial glioma mouse model [137]. However in neuroblastoma, in pre-clinical studies, co-targeting of retinoid and TGFβ signaling pathways through combination of retionoic acid and Kartogenin (TGFβ signaling activating molecule) has decreased the viability of MYCN amplified neuroblastoma cells. CAFs also express different molecules including MMPs and fibroblast activated protein (FAPs), which can be targeted for immunotherapy [136,138,139]. The over-expression of FAP has been associated with tumor incidence and microvessel density in various experimental mouse models [140]. RO6874281 is a bispecific IL2 immunocytokine which targets cancer associated fibroblasts via binding to FAP and has shown potent anti-tumor activity in melanoma, neuroblastoma and colon carcinoma models [141]. Two clinical studies utilizing FAP specific monoclonal antibodies viz. Iodine 131-labeled FAP specific monoclonal antibody (I^131^ F19 MAb) and humanized F19 monoclonal antibody (sibrotuzumab) have been conducted [142]. FAP targeted vaccines have also been explored by several groups, and studies have shown that DNA vaccine targeted against FAP suppresses tumor growth in different cancer models [143,144,145]. FAP targeted vaccines or FAP specific monoclonal antibodies have not been evaluated in neuroblastoma yet but can be used in future clinical trials.

#### 3.4.2. Targeting Mesenchymal Stromal Cells

Mesenchymal stromal cells (MSCs) are also an important component of TME of NB [146]. These are multipotent cells that can be differentiated into different lineages including bone, skeletal muscles, tendon and cartilage [147]. The role of MSCs in neuroblastoma tumor progression and metastasis is well documented [32,148,149]. These cells interact with the tumor cells and other stromal cells to mediate tumor progression. Several reports suggested that these cells can modulate TME by affecting immune cell responses [32,146]. A study by Ma et al. has shown that neuroblastoma cells express both receptors for stromal cell derived factor 1 (SDF 1), i.e., CXCR4 and CXCR7. Furthermore, authors have shown that MSCs can enhance tumor metastasis by secreting SDF1 and this effect can be blocked by AMD3100, an antagonist of SDF-1 [149]. In a separate study, authors have shown that MSCs can upregulate CXCR4 expression and can induce invasiveness in neuroblastoma cell lines [150]. The expression of CXCR4 and CXCR7 has been demonstrated in most of the cell lines derived from patients harboring MYCN amplified or non-MYCN non-amplified tumors [151,152]. The expression of CXCR4 correlated with the bone marrow metastasis in primary neuroblastoma tumors [153]. Pelizzo et al. isolated and characterized MSC from tumor tissues of seven pediatric neuroblastoma patients [32]. The gene expression profiling and the functional properties revealed that these stromal cells contribute to tumor immune escape and metastatic traits of neuroblastoma. DeClerk and his group has done extensive studies on MSC in NB [154,155,156,157]. A recent study by his group has shown that MSCs share characteristics with CAFs and preliminary mouse experiments have suggested that MSCs are recruited into the tumors and are converted into CAFs [155,158]. Several studies have shown that targeting MSCs can be an effective strategy to control tumor growth as these cells can be easily modified to secrete immunomodulatory molecules [159,160]. A recent study by Relation et al. has shown that intra-tumoral delivery of interferon γ-secreting MSCs can polarize macrophages into the M1 phenotype and can suppress neuroblastoma proliferation [161]. To target MSCs, CXCR4 antagonist, Plerixafor has been used for hematopoietic stem cell mobilization in patients with metastatic neuroblastoma [162,163]. A phase 2 clinical trial of Plerixafor in combination with the standard regimen for stem cell mobilization was initiated in patients with refractory neuroblastoma (NCT01288573).

### 3.5. Targeting Infiltrating Immune Cells

The speed of tumor growth depends on the interplay between cancerous cells and the host immune system. The concept of “cancer immunoediting” given by Schreiber et al. consists of three sequential phases: Elimination, equilibrium, and escape [164]. During the initial “elimination phase”, both innate and adaptive immune system work together to eradicate the tumor before it is clinically visible. The immune cells involved in this phase are effector CD8+ T cells, NK cells, macrophages, dendritic cells, and natural killer T cells. Most tumor cells are destroyed in the equilibrium phase, however, some rare mutant cells are not destroyed in this phase and they enter into next “equilibrium phase.” In the “equilibrium phase” tumor cells are maintained in a state of immune-mediated dormancy and this stage may last for the life-time of an individual [165]. The length of the equilibrium phase depends on the stability between the immune tolerance of tumor cells and the strength of endogenous anti-tumor immunity. This process of continuous immune pressure on genetically unstable tumor cells leads to the generation of variant tumor cells, which are no longer been recognized by the immune system and enter into the “escape phase”, in which tumors begin to grow progressively without any immunological constraints and establish immunosuppressive microenvironment [166]. The role of tumor infiltrating leukocytes in mediating anti-tumor immunity and in the modulation of TME has been demonstrated in various studies, including neuroblastoma [167,168]. During early neoplastic lesion, the infiltration of cytotoxic CD8+ T cells dominates, however as the tumor progresses, these cells are outnumbered by tumor associated macrophages, myeloid derived suppressor cells and regulatory T cells which mediate immunosuppression in TME [167,168]. Overall, the presence of cytotoxic CD8+ T, CD4+ Th1 cells and NK cells serve as a prognostic factor of favorable outcomes in various solid cancers including NB [169]. On the contrary, the presence of immunosuppressive cells like TAMs, MDSCs, and T_reg_ hinder effective anti-tumor immune responses and thus may be associated with poor clinical outcome in NB [28,170].

#### 3.5.1. Myeloid Derived Suppressor Cells

Myeloid-derived suppressor cells (MDSCs) are immature myeloid cells that fail to differentiate into macrophages, granulocytes and dendritic cells, but expand in pathophysiological conditions like inflammation and cancer [171,172]. MDSCs play an important role in mediating immunosuppression in TME as these cells suppress the activity of T cells, NK cells, and dendritic cells [171,173]. In addition to depleting L-arginine, a factor important for T cell proliferation, MDSCs also produce nitric oxide and reactive oxygen species which affect T cell function [174]. MDSCs also produce cytokines, including IL-10 and TGF-β, to induce Treg cells, and inhibit NK cell activation and cytotoxicity [171,174]. The strategies that are aimed to block its accumulation, recruitment, and reversal of MDSC-mediated immunosuppression are in clinical trials for various solid tumors [175,176]. There is a limited availability of the literature on the clinical significance of MDSCs in NB. Gowda et al. have shown that MDSCs suppress adaptive immune responses in low-risk NB patients [177]. The accumulation of MDSCs was also reported during tumor progression in the TH-MYCN driven mouse model, and the treatment of low dose of aspirin is reported to reduce tumor volume and decreased infiltration of MDSCs in this mouse model [167,178]. This study has shown that treatment of low dose of aspirin reduced tumor burden, decreased the presence of cells of innate immune system, M2 macrophage polarization and intratumoral expression of TGFβ, thromboxane A2 and prostaglandin A2 in the TH-MYCN driven mouse model. In another study, Santilli et al. have shown that polyphenol E, a clinical grade mixture of green tea catechins inactivates MDSCs and promotes anti-tumor immune responses in both, transgenic TH-MYCN mouse model and A/J mouse implanted with Neuro2a cells [179]. In a recent study, using the TH-MYCN mouse model, Mao et al. has shown that targeting immunosuppressive myeloid cells can potentiate checkpoint blockade in NB [170]. In another study, gene modified NK cells (NKG2D.ζ) was generated by fusing NKG2D to the cytotoxic ζ-chain of the T-cell receptor. This study has shown that NKG2D.ζ-NK cells are cytotoxic against MDSCs and work effectively in eliminating immunosuppressive tumors [180]. Various ongoing studies in in vivo mouse models have shown that targeting MDSCs enhances anti-tumor immune responses in NB [179,181], suggesting that MDSCs may play roles in cancer related inflammation to enhance NB progression. Various strategies to either block accumulation of MDSCs or recruitment of MDSCs or polarization of MDSCs into immunosuppressive phenotype are in use and molecules targeting these strategies, including all-trans retinoic acid (ATRA), bevacizumab, CCX9588, tadalafil are in clinical trials for various cancers and has been reviewed before [175]. Various Phase II clinical trials of ATRA in combination with GMCSF and 3F8 mAb are in clinical trials for NB patients (NCT01183429, NCT01183884, NCT01183416, NCT01183897, and NCT00969722). Also the reduction of MDSCs by ATRA improves the efficacy of CAR therapy for NB [182]. Bevacizumab has also been evaluated in various phase I and II clinical trials for NB and has been discussed before in this review article.

#### 3.5.2. Regulatory T cells (Treg)

Tregs are highly immunosuppressive fractions of CD4+ T-cells and are known to play a major role in maintaining self-tolerance, immune homeostasis and preventing autoimmunity. Treg cells exhibit their suppressive activity through several mechanisms, including inhibition of antigen presenting cell (APC) maturation through the CTLA-4 pathway; secretion of inhibitory cytokines such as IL10, TGF beta, IL35; and expression of granzyme and perforin which kills effector T-cells. Various reports have shown that the accumulation of Treg infiltrated in tumor tissues is associated with worse prognosis in various cancers. Several potential therapies target Treg cell suppression either directly or indirectly including candidates targeting CD25, CTLA-4, OX-40, GITR, and CCR4 [175]. The role of Treg in the progression of NB is highly controversial. Very limited studies have shown an association between Treg frequency and clinical outcomes in NB patients. Some studies have shown an increased number of circulating regulatory T cells in NB patients, as compared to healthy individuals, but did not correspond to prognostic factors [183,184]. In another report lower frequency of Treg population has been observed in the bone marrow and peripheral blood samples of NB patients [185]. Although there are several inconsistencies in the data related to the presence of Treg and clinical outcome, preclinical data generated in mouse models indicates that in vivo depletion of Treg increases the efficacy of immunotherapy mediated by CD8+ T cells in vivo [186,187,188]. One study by Jing et al., has shown that the deletion of CD4+T cells enhances the immunotherapy of neuroblastoma [189]. In another study, it was shown that low expression of CD4+/CD25+/CD127- T reg cells and high levels of IFNγ are associated with improved survival of neuroblastoma patients which are treated with anti-GD2 antibody ch14.18/CHO in combination with interleukin 2 (IL2) [190]. Recent pieces of evidence have suggested that indoleamine 2, 3-dioxygennase (IDO) activity is critical for the activity of FoxP3 T_reg_ cells and various IDO inhibitors, including epacadostat, indoximod, are used in pre-clinical and clinical studies for various cancers other than neuroblastoma [191]. Although, it is important to mention here that all the clinical trials related to IDO inhibitors have been halted as Epacadostat has shown negative results in Phase 3 clinical trials in combination with nivolumab and pembrolizumab [192].

#### 3.5.3. Macrophages

Macrophages are the most abundant and highly plastic immune cell infiltrate found in solid tumors. The presence of macrophages is correlated with worse prognosis in various solid tumors including neuroblastoma [28,193]. Macrophages can be classified into two main populations in the TME based on their gene expression profiles [194,195,196]. In the presence of lipopolysaccharide (LPS) or IFN gamma, macrophages are polarized into the M1 phenotype, which produces immunostimulatory cytokines and exhibits tumor suppressive activities. On the contrary, M2 polarized macrophages are activated by IL4 and are known to promote tumor growth, angiogenesis, and immunosuppression [197]. M2 polarized macrophages are commonly known as tumor associated macrophages and they express M2 macrophage markers, i.e., CD163 or CD206, secrete vascular endothelial growth factor (VEGF), matrix metalloproteinase (MMP) and produces immunosuppressive cytokines, i.e., IL10, and transforming growth factor β (TGFβ) which dampens effective anti-tumor immune responses and promote tumor progression and metastasis. In addition to IL-4 or IL-13, B cell derived immunoglobulins have been shown to accumulate in pancreatic adenocarcinomas and squamous cell carcinomas and to stimulate the activation of macrophages via engagement with FcγR receptor [198,199].

Various shreds of evidence suggest that TAMs can facilitate the progression of neuroblastoma [28,200]. Seeger and group have focused most of their studies on non-MYCN amplified tumors and showed that these tumors express high level of inflammatory genes related to macrophages [26,28,201]. These studies have identified gene signature comprising of IL6, IL10, and TGF-β which was associated with a dismal prognosis. This group also detected CD68 positive TAMs expressing IL6 in the metastatic bone marrow samples. Asgharzadeh et al., has reported that metastatic tumors had higher infiltration of TAMs, as compared to loco regional tumors and the presence of CD163+ TAMs was associated with worse prognostic signature [28]. Moreover, they reported that patients with an age of ≥18 months have higher expression of TAM related genes, including CD14, CD16, CD33, Il-10 and IL6R as compared to patients diagnosed at the age of ≤18 months. A study by Ara et al. has shown that MSC and TAMs are major sources of IL6 in neuroblastoma TME [202]. In another report, Hadjidaniel et al. suggested that TAMs promote neuroblastoma tumor growth via the up-regulation of c-myc [203]. To overcome the immunosuppressive or pro-tumoral functions of TAMs, current therapies are mainly focused on; 1) blockade of macrophage recruitment, 2) depletion of existing macrophages, or 3) reprogramming of macrophages into anti-tumor phenotype [175].

The inhibition of CCL2 with various antibodies is known to block the recruitment of macrophages in the TME [204,205,206]. Anti-CCL2 antibody carlumab was well tolerated and showed great efficacy in various solid cancers [204,205], and has not yet been tested in neuroblastoma models. CSF-1 receptor is expressed by most of the cells of the monocytic lineage and is a direct target to block monocytic precursors directly and indirectly. Antagonists or antibodies to CSF1R have been developed and tested in various preclinical models (e.g., cervical cancer, pancreatic cancer, and glioblastoma) in combination with chemotherapy, radiation therapy, and checkpoint inhibitors, whereby they depleted immunosuppressive macrophages and increased the CD8/CD4 ratio in the tumors [207]. A recent report by Seeger suggested that blockade of the CSF1 receptor improves the efficacy of chemotherapy in neuroblastoma in the absence of T lymphocytes [201]. In another study, Mao et al. has shown that infiltrating CSF1R positive myeloid cells predict poor outcomes in neuroblastoma and targeting myeloid cells with CSF1R inhibitor BLZ-945 alone or in combination with anti-PD1 antibodies improves survival in TH-MYCN mouse model [170]. Various CSF1R inhibitors, including BLZ-945, RG7155, either alone or in combination with checkpoint inhibitors are in clinical trials for various solid cancers or has been reviewed previously [175]. Repolarization of macrophages from the immunosuppressive phenotype into the immunostimulatory phenotype has been emerged as an effective strategy to control tumor growth. Various preclinical studies identified signaling pathways or key genes, such as the jumonji domain, containing proteins (JMJD3), STAT3, STAT6, BRD4, Myc, Rac2, Syk, PI3Kγ, Btk, etc. which play a crucial role in stimulating alternative activation of macrophages and promoting tumor growth in solid tumors [115,120,208,209,210,211]. The role of Rac2 in blocking macrophage differentiation in MYCN driven mouse model of neuroblastoma has been reported before [208].

The research publications from our group have demonstrated that SF1126 and SF2523 can polarize immunosuppressive macrophages to immunostimulatory phenotype and these inhibitors can simultaneously block tumor proliferation and can activate adaptive immune responses [114,115,120]. My recent work has shown that novel dual Syk-PI3Kγ inhibitor SRX3207 effectively relieves tumor immunosuppression in solid tumors [211] and has shown great efficacy in targeting immunosuppressive TME of NB (data unpublished). Sondel group has done intensive research on the repolarization of macrophages by the use of monoclonal CD40 mAb and cytosine-phosphate-guanosine containing oligodeoxynucleotide 1826 (CpG-ODN) (IT) in neuroblastoma models [212,213,214]. Their studies have shown that the combination of IT with cytotoxic chemotherapy provides synergistic anti-tumor effects in the neuroblastoma mouse model [215]. Recently Sondel group has shown that CD40 mAb along with CpG and anti-CTLA4 antibody provides potent anti-tumor immune responses in immunologically cold murine syngeneic neuroblastoma murine model [214].

## 4. Conclusions and Future Directions

In summary, basic research on the stromal and immune cells of NB TME is crucial to develop novel therapeutics for this childhood cancer. The immunosuppressive microenvironment is predominant in NB tumors and hence strategies to target immunosuppressive immune cells, like macrophages and MDSCs should be carefully considered for development of therapeutics. The checkpoint inhibitor therapy has shown great success in other solid cancers but in NB these inhibitors have not shown any significant benefit. Hence, combining TME targeting strategies with checkpoint inhibitor therapy might provide some benefit in this childhood malignancy. In this review, I have summarized different components of NB TME which can be targeted for the development of therapeutics. I have also highlighted on the dual PI3K/BRD4 inhibitory chemotypes SF1126 and SF2523 which can concomitantly inhibit several tumor promoting signaling pathways and can activate anti-tumor immune response by blocking myeloid cell mediated immunosuppression. These inhibitors in combination with checkpoint inhibitor therapy need further investigation in neuroblastoma models.

## Figures and Tables

**Table 1:** Anti-angiogenic agents used alone or in combinatorial therapy in clinical trials in neuroblastoma.

Therapeutic Agent	Target	Combination Therapy or Drug Used	Clinical Trial and Reference	Status of Clinical Trial	Phase
**Bevacizumab**	VEGF				Phase 1
		Irinotecan plus temozolomide	NCT01114555 [107]		Phase 2
		Cyclophosphamide plus topotecan	NCT01492673		Phase 2
		Cyclophosphamide and zoledronic acid	NCT00885326		Phase 1
		Iodine 131 monoclonal antibody 3F8	NCT00450827 [109]		Phase 1
		Temozolomide plus irinotrecan plus topotecan plus dinutuximab (BEACON)	NCT02308527 [110]		Phase 2
**Pazopanib (GW786013)**	VEGFR1, VEGFR2, VEGFR3		NCT01130623		Phase I
**SF1126**	PI3K/mTOR		NCT02337309 [22]		Phase I
